# Analytical Modeling of a New Compliant Microsystem for Atherectomy Operations

**DOI:** 10.3390/mi13071094

**Published:** 2022-07-11

**Authors:** Pietro Ursi, Andrea Rossi, Fabio Botta, Nicola Pio Belfiore

**Affiliations:** 1 Department of General and Specialized Surgery Paride Stefanini, Sapienza University of Rome, 00161 Rome, Italy; pietro.ursi@uniroma1.it; 2 Department of Industrial, Electronics and Mechanical Engineering, Roma Tre University, 00146 Rome, Italy; andrea.rossi@uniroma3.it (A.R.); nicolapio.belfiore@uniroma3.it (N.P.B.)

**Keywords:** MEMS, PRBM, compliant mechanism, atherectomy

## Abstract

This work offers a new alternative tool for atherectomy operations, with the purpose of minimizing the risks for the patients and maximizing the number of clinical cases for which the system can be used, thanks to the possibility of scaling its size down to lumen reduced to a few tenths of mm. The development of this microsystem has presented a certain theoretical work during the kinematic synthesis and the design stages. In the first stage a new multi-loop mechanism with a Stephenson’s kinematic chain (KC) was found and then adopted as the so-called pseudo-rigid body mechanism (PRBM). Analytical modeling was necessary to verify the synthesis requirements. In the second stage, the joint replacement method was applied to the PRBM to obtain a corresponding and equivalent compliant mechanism with lumped compliance. The latter presents two loops and six elastic joints and so the evaluation of the microsystem mechanical advantage (MA) had to be calculated by taking into account the accumulation of elastic energy in the elastic joints. Hence, a new closed form expression of the microsystem MA was found with a method that presents some new aspects in the approach. The results obtained with Finite Element Analysis (FEA) were compared to those obtained with the analytical model. Finally, it is worth noting that a microsystem prototype can be fabricated by using MEMS Technology classical methods, while the microsystem packaging could be a further development for the present investigation.

## 1. Introduction

For decades, minimal invasive surgery methods have been constantly increasing their fields of applications due to their great advantages with respect to traditional surgeries. For example, minimally invasive endovascular procedures can be conveniently adopted in the case of artery or vein obstructions, by means of balloon angioplasty and percutaneous transluminal angioplasty (PTA). These methods make use of a catheter that dilates the stenosed artery in order to mount a spring-loaded metal stent that expands, allowing the blood to flow regularly.

Sometimes, the obstructing material in vessels can be removed and in these cases, a special filter is used to prevent the occlusion of the peripheral cerebral vessels due to the detachment of small fragments. For example, carotid stenosis surgery [1,2] has been applied to remove plaques from the artery, with the purpose of restoring the normal blood flow. Carotid thromboendoarteriectomy treats stenotic disease with vessel occlusion between 70% and 99%, and implies usually a 10 cm incision in the patient’s neck to reach the diseased artery and remove the plaque.

Some alternatives to conventional endovascular angioplasty have been developed for the treatment of peripheral artery disease. Among these, atherectomy offers the possibility to minimize the stretch damaging on arterial walls and to reduce the risk of restenosis. Several atherectomy devices have been deployed.

- Directional atherectomy [3,4,5,6] uses a side-cutting rotating blade, with or without a preloaded distal flush tool, with or without collecting nosecone, with or without active aspiration, with or without OCT guidance, with or without an apposition balloon (see for example SilverHawk^®^, TurboHawk^®^, HawkOne^®^ by Medtronic, Minneapolis, MN, USA, or Pantheris^®^ by Avinger Inc., Redwood City, CA, USA);
- Rotational atherectomy [7,8,9,10,11,12,13] uses a rotating olive-shaped burr, with single or multiple blade sets, that removes plaque microparticles by means of abrasive diamond chips embedded in the rotor, with active debris aspiration or mechanical removal (see, for example, Pathway Jetstream PV^®^, Peripheral Rotablator^®^ by Boston Scientific, Boston, MA, USA or Phoenix^®^ by AtheroMed Inc., Menlo Park, CA, USA or Rotarex^®^S by Straub Medical, Wangs, Switzerland));
- Orbital atherectomy [14] uses an eccentric diamond-coated crown with atherectomy depth increasing with speed (see, for example, Diamondback 360^®^ by Cardiovascular Systems Inc., St. Paul, MN, USA);
- Crosser Chronic Total Occlusion recanalization systems [15] use high-frequency mechanical vibrations that are transmitted to a metallic tip with a saline flush cooling system. (see, for example, Bard Peripheral Vascular Inc., Tempe, AZ, USA, Crosser peripheral CTO recanalization systems);
- Excimer Laser atherectomy [16,17,18], uses ultraviolet radiation to remove atheroma (see for example Turbo-Tandem, Turbo-Elite and Turbo-Power catheters by Spectranetics-Philips, Eindhoven, The Netherlands)

The present work offers a possible alternative to the above-mentioned systems for atherectomy, with the purpose of minimizing risks for the patient and maximizing the cases for which the system can be used, due to the limited dimensions of the lumen.

The development of this new device required a very interdisciplinary approach and so different know-how’s were required. The initial part of the engineering idea will be herein described. More specifically, this paper is dedicated chiefly to the design and optimization problem, while prototyping, packaging and test will be left as further developments, to bring the device to a higher levels of technological readiness.

Considering fabrication and testing, a few words can be herein spent to anticipate that the proposed device can be fabricated in several ways. However, for endoluminal application and atherectomy surgical operation, it is necessary to push miniaturization down to one millimeter and even less. Therefore a real prototype can be hardly fabricated with the ordinary technological machining operations. For this reason, it is necessary to resort to more sophisticated methods, indeed, those based on nanotechnologies. All the MEMS Technology-based procedures can be adopted to fabricate the real device. The adopted design method is compatible with MEMS Technlogy-based processes, in particular with the deep reactive-ion etching (DRIE) process [19,20], applied to a SOI (Silicon-on-Insulator) wafer and described in detail in ref. [21]. Therefore, the results obtained by means of this method can be physically implemented at the micro- or even nano-scale. Thanks to the versatility of the above-mentioned method, many compliant micromechanisms can be implemented in a wide range of applications such as Lab-on-Chip or medical diagnosis devices [22,23,24]. In the case under study, an additional challenge arises for the project that consists in the adoption of a new packaging technique that prevents fluids from getting inside the compliant mechanism. Fabrication, testing and packaging will be discussed in a forthcoming dedicated contribution, while the present paper paves the way to them, thanks to modeling and simulation. In fact, considering design and optimization, the present paper offers a description of the design method and of its specific application.

In order to better explain how the method herein proposed and its application to the special task of atherectomy convey some new elements in the literature, it is convenient to position the present work within the general framework of the Research in Compliant Mechanisms.

Actually, the present paper owes much to the pioneering contributions which in the literature introduced the concept of “compliant mechanism”. Since 1975 [25], flexural mechanisms with large static deflections have been proposed as function generators. Nonlinear analysis has been suggested via Finite Element Analysis, while the problem of synthesis has been solved by means of optimization techniques. In 1980, the compliant mechanisms were proposed to provide machines self-correcting motions and adaptable outputs [26]. In 1987, some fundamental kinematic properties of a compliant mechanism were identified and discussed, developing a terminology specifically appropriate for this class of mechanisms, such as the degrees-of-compliance (or compliance number) and type synthesis [27]. Moreover, a standard nomenclature was suggested in 1994 [28] and the advantages of the compliant mechanisms were underlined in terms of the fewer parts required and less noise, wear and backlash than their rigid-body equivalent mechanisms.

A great impulse to the design of lumped compliance mechanisms was due to the concept of the pseudo-rigid-body model (PRBM) that proved to be good to approximate more complex geometry and nonlinearities [29]. The concept of equivalent spring stiffness was also investigated by modeling the force vs deflection relations of large-deflection members [30]. Since then, various models have been proposed to facilitate the analysis and synthesis of compliant mechanisms, with one or more degrees of freedom [31,32,33,34].

The approximation offered by the PRBM has been further improved by a novel method that allows designers to implement classical algorithms of functional design at the microscale with more accuracy and confidence in results. Thanks to this method, it is possible to find solutions to a large class of applications formulated as problems of synthesis of plane mechanisms. More specifically, the above-mentioned procedure consists in the application of the kinematic synthesis to an ordinary mechanism [35], namely the PRBM, followed by the identification of a compliant mechanism obtained by applying the joint replacement method [36,37] to the PRBM designed in the previous stage. The joint replacement step is performed by making use of the CSFH (conjugate surfaces flexure hinge) [38]. The approach has been used to obtain different multi-DoF, multi-hinge devices, such as 3-DoF platforms [39,40] and microgrippers for biosample characterization [41,42,43,44,45] that have also been considered for use in a surgical scenario [46,47,48].

The present investigation introduces a recently invented microsystem for atherectomy treatments [49], with the aim of fabricating a new compliant micro-lancet with prescribed kinematic and static characteristics. Based on topological and kinematic synthesis, a new PRBM has been identified from the Stephenson kinematic chain. Then, the joint replacement methods transformed the PRBM into a new mechanical structure has been derived, that consists of a compliant mechanism (CM) with lumped compliance, two loops and six elastic joints. As described in the next section, the development of kinetostatic modeling needs to involve calculation of the stiffness of the flexible elements and the elastic energy stored in the hinges (see Equation (29) in the next section) and so, given the complexity of the multi-hinge and multi-loop microsystem under analysis, a new approach had to be conceived using the results of the preliminary inverse static problem. Moreover, complementary energy due to the PRBM–CM replacement was introduced and calculated. These ideas make the adopted approach quite different from those already presented in literature. Thanks to this method, some performance indices could be calculated, such as, for example the mechanical advantage (MA) for compliant mechanisms [50,51]. Furthermore, closed-form solutions are obtained for the problems of position and first-order kinematics, from which a symbolic expression of the mechanical advantage of the microlancet is derived.

The resulting hinge stiffness, elastic energy values and the MA index can be compared to the ones obtained by means of FEA simulations, in order to check the analytical findings.

## 2. Development of the New Microsystem

This section is dedicated to the description of the method adopted to design and evaluate a multi-loop lumped compliant mechanism. Firstly, the design approach is based on classical stages of mechanism design, whose result consists of the PRBM. Then, the rigid-body replacement method and inverse static analysis are carried out to complete the design phase and evaluate the performance of the obtained compliant mechanism.

### 2.1. Topological Synthesis

From the functional requirements, a suitable kinematic chain (KC) is obtained. Then, one of the links of the KC must be chosen as the frame link and the kinematic pairs have to be labeled. Graph theory has been conveniently used in mechanism science to represent the topological characteristics of a mechanism. According to this method, links and pairs correspond to nodes and edges of a graph, respectively. The topological characteristics are useful for several reasons, such as evaluation of degrees of freedom, number of independent loops, adjacency and the incidence matrices [52], and are helpful to solve problems of kinematic, static and dynamic analysis. Then, an ordinary mechanism is selected.

### 2.2. Kinematic Analysis and Synthesis

Once the ordinary mechanism has been obtained, the classical methods of kinematic analysis and synthesis can be applied to obtain a particular mechanism with specific dimensions of the links. Furthermore, the mechanical advantage or other relevant parameters can be determined at this stage.

### 2.3. Rigid-Body Replacement Method

The previous classical phases of design yield an ordinary mechanism that, for the proposed method, corresponds to the PRBM. The latter can be transformed into a compliant mechanism by means of the rigid-body replacement method, which leads to the final compliant mechanism. The method consists of replacing the kinematic pairs of the PRBM with elastic elements, or flexures. The obtained compliant mechanism, being a monolithic structure, can be fabricated both with traditional and MEMS-based processes. Generally, from the PRBM, several different compliant mechanisms could be generated, depending on the replacement criteria and on the type of flexible element chosen for the replacement step.

Although the stage of kinematic synthesis does not deal with the material properties, the load types, the residual stress, the minimum dimensions of the components and the temperature effect, these properties may affect the following stage of designing the compliant mechanism from the PRBM by means of the joint replacement method. Generally, this transformation is made by assuming that the material is homogeneous and isotropic. Actually, these two properties do not perfectly apply to an SOI (Silicon on Insulator) wafer. In fact, as far as homogeneity is concerned, the SOI wafer is composed of three layers that are parallel to the plane of motion and so they are not homogeneous in the transverse direction but they present a similar behaviour along the plane of motion. As for isotropy, silicon presents different characteristics along different directions. Nevertheless, from the experimental evidence from previous experience, considering the material as homogeneous and isotropic does not imply, generally, a significant error. As far as the load types are concerned, the developed model assumes that forces are concentrated to the lancet tip and on the input link. Within the limits of the silicon mechanical properties, a linear behaviour can be assumed to model the material with no problems. Similarly, the effect of residual stresses can be excluded during the normal working of the mechanism, while they will affect very much the fabrication process. The minimum dimensions of the components usually do not much affect the CM functionality, although previous investigation [53] has shown that DRIE (Deep Reactive Ion Etching) may induce scalloping on the structure that allowed stress concentrations to be able to halve the stress resistance in the elastic hinges. Finally, the temperature effect is very limited in the microsystem because there are no heating sources and the thermal expansion coefficients from silicon do not much affect its functionality.

In the present investigation, among many possibilities, the compliant mechanism is obtained by replacing a revolute pair with a constant-curvature beam, positioning its center of elastic weights on the center of the revolute joint [54]. Therefore, the rigid-body replacement step is performed by substituting all the PRBM revolute joints with the same flexure, and by following the same replacement criterion. This premise determines many advantages from the fabrication and from the design points of view, as will be made clear in the next sections.

### 2.4. Inverse Static Analysis

Once the compliant mechanism is obtained, it is necessary to analyze its static behaviour. For this purpose, the inverse static problem of the PRBM can be solved to derive the reactions on each elastic joint and the stiffness of the flexure elements. Since all the kinematic variables have already been derived at the design stage, if the external forces are known, the joint reactions and stiffness can be calculated for a specific mechanism configuration by solving a system of linear algebraic equations. However, the internal torques $\tau_{ij}$ between link *i* and *j* depend on the stiffness

(1) $$k_{ij}=\frac{EI}{L}$$

of each flexible element, where *E* is the Young modulus, *I* is the second area moment of the flexure cross-section about the bending axis and *L* is the flexure axis length.

It is worth noting that Equation (1) is only an approximation. In fact, nanotechnology-based processes may have some implications on the geometry and on the materials of the mechanical structure. For example, using a metal hard mask and DRIE process [21] would imply a metal deposition on a silicon substrate, with the result of achieving a sort of composite beam.

**Figure 1:**
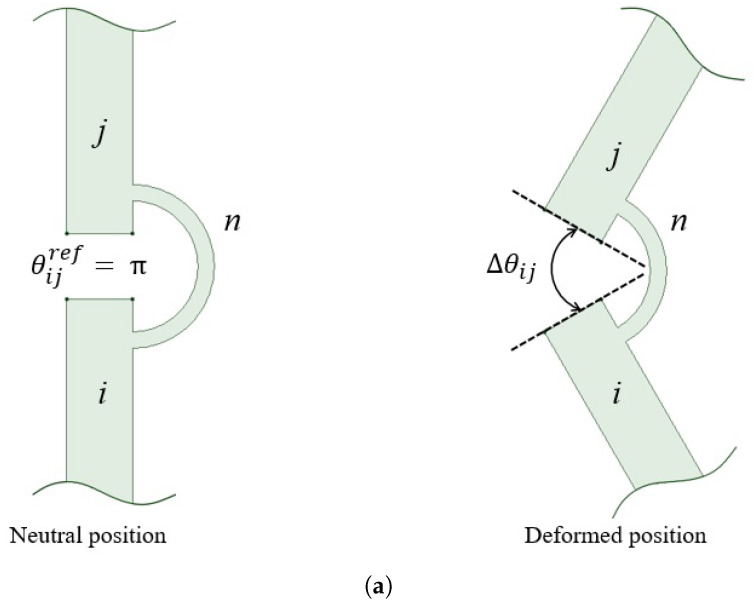

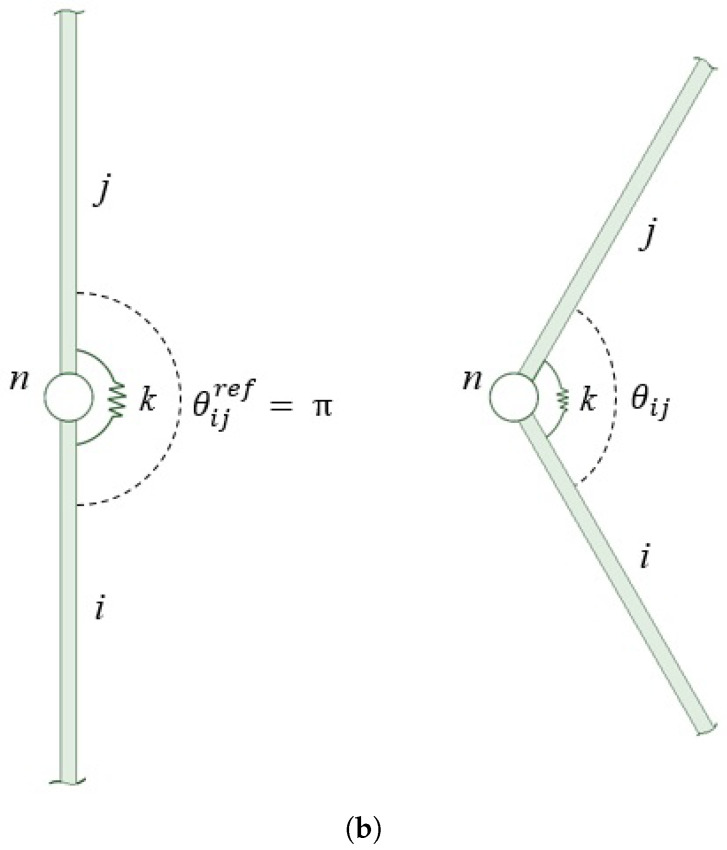
A representation of the *n*-th flexure hinge (**a**) and its PRBM schematization (**b**).

Since, as supposed in Section 2.3, all the flexure hinges have equal geometry and materials, the torque exerted by the *n*-th flexure hinge on each of the revolute links *i* and *j* can be written as (see Figure 1)

(2) $$\tau_{ij}=-k_{n}\Delta \theta_{ij}=-k\Delta \theta_{n}$$

where $\Delta \theta_{ij}=\Delta \theta_{n}=\theta_{ij}-\theta_{ij}^{ref}$ and $\theta_{ij}^{ref}$ is the reference angle corresponding to the neutral position of the PRBM. Therefore, *k* can be regarded as a unique unknown variable in the inverse dynamic problem, so that the vector of the unknowns include the components of the reactions exerted by the kinematic pairs and the stiffness constant *k*.

### 2.5. Elastic Energy Evaluation

As known, the application of the principle of virtual works in static condition is a straightforward means to evaluate the amounts of the input and the output forces in ordinary mechanisms. Unfortunately, in compliant mechanisms, the input to output power, at a balance configuration, can be invoked no more, because they require a certain overall energy $U_{O}$ to reach a final deformed configuration. In this investigation, assuming that no deformation energy is stored in the rigid bodies, the total required deformation energy will be regarded to be the sum of the elastic energy $U_{R}$, due to the relative rotations between the rigid links, plus the elastic energy $U_{E}$, due to the rigid-body replacement,

(3) $$U_{O}=U_{R}+U_{E}\;.$$

In Equation (3), $U_{R}$ is calculated by making reference to the rotation angles that apply to the revolute hinges of the Pseudo Rigid Body Mechanism (PRBM) for a given configuration. Hence, considering the total energy absorbed by the revolute joints (equipped with a torsional spring *k* ), it is assumed that $U_{R}=U_{PRBM}$, with

(4) $$U_{PRBM}=\frac{k}{2}\sum_{n=1}^{N}\Delta \theta_{n}^{2}\;,$$

where *N* is the total number of flexure elements.

**Figure 2:**
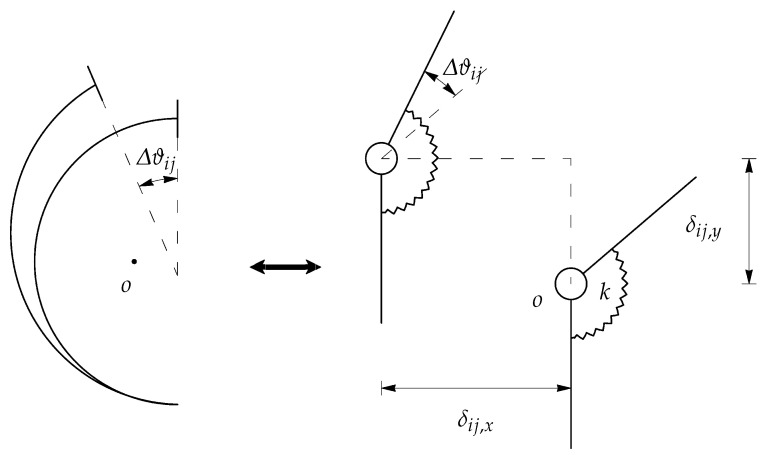
Rigid-body replacement and axis drift.

The term $U_{E}$ is calculated as the energy quota that is adsorbed by the real compliant mechanisms due to the parasitic motion of the centers of the relative motion between two adjacent links (absent in ordinary revolute pair). In other words, during the rigid-body replacement step, the centers of the pin and the sleeve of each revolute joint are assumed to be coincident, regardless of the mechanism configuration. Unfortunately, this is not the case for the corresponding compliant mechanism, and so this affects unwanted axis drift. Figure 2 shows the rigid-body replacement step, highlighting the center of the elastic weights *o* of the constant-curvature beam and the displacement of the revolute joint.

**Figure 3:**
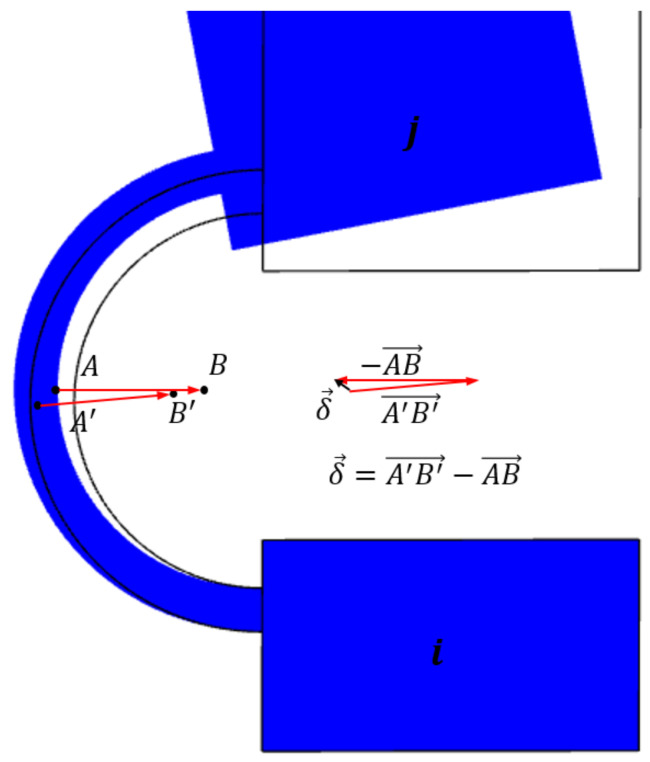
Difference vector of the distance between points A and B of a generic flexure hinge calculated, respectively, for the neutral and deformed configuration.

In order to evaluate this contribution in terms of stored energy, a hybrid procedure is used. Firstly, the centering errors of any single revolute joint in the PRBM is evaluated by means of FEA for the assigned values of the input and output forces (I). Then, the reaction forces acting in correspondence with the PRBM joints are evaluated by means of inverse dynamic (II).

(I) The first task can be explained with reference to Figure 3, which shows the relative difference $\bar{\delta }$ of the vectors $\vec{AB}-\vec{A^{\prime }B^{\prime }}$ before and after the deformation, respectively. *A* is the centerpoint of the curved beam midsection, *B* is the center of the elastic weights when the neutral configuration is considered. $A^{\prime }$, $B^{\prime }$ are the corresponding points once the deformed position is achieved. Vector $\bar{\delta }$ can be evaluated for each flexure (corresponding to revolute joints), giving rise to a good estimation of the centering errors.
(II) The second task is simply carried out by calculating the reaction forces $F_{ij}=-F_{ji}$ at the *i*-*j* revolute pair, and their *x* and *y* components, $F_{ij,x}$ and $F_{ij,y}$, respectively, by means of a simple inverse dynamic analysis of the PRBM.

Neglecting the energy dissipated due to friction and considering a linear elastic material, the work of a force gradually applied to an elastic system is half of the work calculated as the product of the final balance value of the force by the final displacement, according to Clapeyron’s theorem. Then, the elastic energy $U_{E}$, due to the joint replacement approximation, is given by

(5) $$U_{E}=\frac{1}{2}\sum_{i<j}F_{ij,x}\delta_{ij,x}+\frac{1}{2}\sum_{i<j}F_{ij,y}\delta_{ij,y}$$

where $\delta_{ij,x}$ and $\delta_{ij,y}$ are the *x* and *y* components of ${\bar{\delta }}_{ij}$, respectively.

Finally, $U_{O}$ can also be calculated by using Finite Element Analysis (FEA), which simulates the deformation experienced by the compliant mechanism. The numeric procedure implemented by FEA offers directly the total amount $U_{FEA}$ of energy required to deform the entire structure. In this investigation, it is assumed that $U_{O}=U_{FEA}$.

### 2.6. Stiffness Evaluation

The elastic energy $U_{FEA}$ also includes the contribution due to the deformation of the links. This amount can be neglected because the links behave approximately as rigid links. Furthermore, it also includes the amount $U_{E}$, which is generally one order of magnitude less than $U_{R}$. Therefore, an approximate expression of the revolute joint stiffness

(6) $$k_{FEA}=\frac{2U_{FEA}}{\sum_{n=1}^{N}\Delta \theta_{n}^{2}}$$

can be obtained, where $\Delta \theta_{ij}$ are also calculated by using FEA results, as shown in Figure 1.

In this work, the considered microsystem consists of a compliant mechanism designed with the requirement to be miniaturized by using MEMS Technology-based processes. The purpose of the application involves the use of the microdevice for endoluminal surgical scenarios, such as the plaque removal from the lumen without perforating the inner walls. Lumped compliance was adopted to build six selective compliance elements that connect six rigid bodies. It is worth noting that there is also a seventh kinematic pair that consists of a prismatic pair which is composed of the input cable or moving link that translates with respect to the shell (frame link).

**Figure 4:**
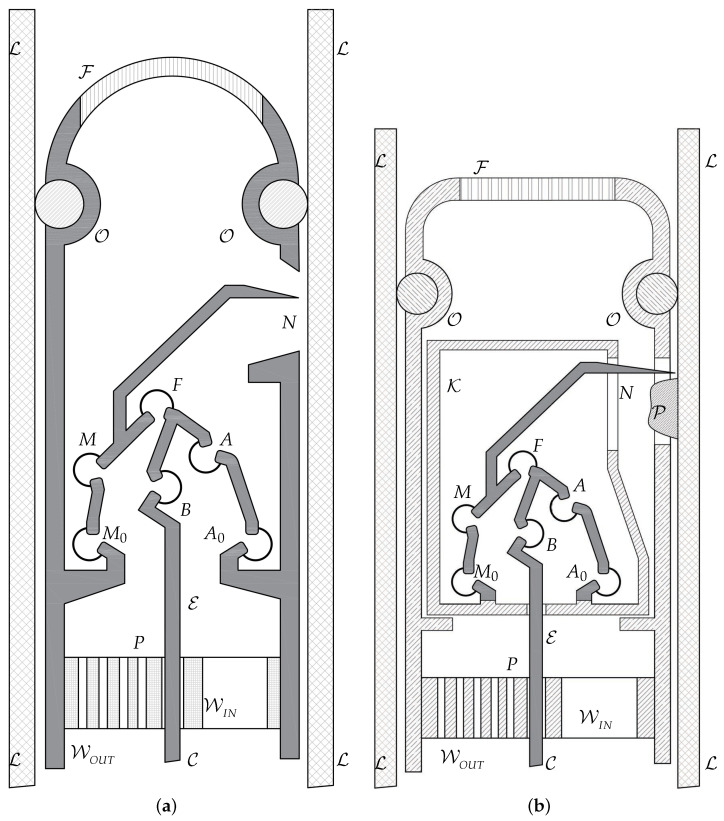
A view of the endoluminal microsystem designed for future validation at TRL 4 (**a**) and TRL 5 with the packaging K (**b**).

Two possible configurations are illustrated in Figure 4, where the compliant mechanisms can been downsized in such a way as to fit the lumen. Since the geometry downsizing is restricted only by the fabrication constraints, the overall geometry size can span from 2 mm to 15 mm, depending on the application.

Figure 4a shows a possible configuration for endovascular surgery applications. The layout is intended to be used at technology readiness level (TRL) 4, which implies the validation of the device components in the laboratory. For this reason, this layout is illustrated without the packaging case. The image represents a cross-sectional view of a lumen L where the head of a catheter has been introduced. An O-ring O is mounted for sealing the fluid and for preventing any embolus to travel in the arterial flux. Furthermore, a filter F is positioned on the top of the capsule for the same reason, without blocking the normal blood flow. The six elastic joints can be designed either as flexible beams with circular axis or as conjugate surface flexure hinges (CSFHs) [38,55,56], and are represented in the figure by the centers $M_{0}$, *M*, *F*, *B*, *A* and $A_{0}$ of the elastic joints, whereas *P* represents the seventh kinematic pair that consists of a prismatic pair used to pull the input element E attached to the remote control cable C.

Figure 4b shows the layout that is intended to be used later, at TRL 5, which implies the validation of the device in the real application. In this case, the mechanism is embedded within the packaging case K. The system is designed in such a way that the tip point *N* moves parallel to the lumen walls. This mobility constraint, that is, the required kinematic condition for the design, prevents possible damage to the wall tissues, while it is particularly convenient to remove obstructing plaques P.

The removing efficacy of the lancet tip *N* could be supported, depending on the application, by an electric current or by the chemical action of drugs provided to the tip by a remotely induced flow. In Figure 4, the inlet and outlet channels are represented by the ducts $W_{{}_{IN}}$ and $W_{{}_{OUT}}$, respectively.

### 2.7. Topology

With reference to Figure 4, the tip point *N* of the lancet must have a velocity that is parallel to the lumen walls L. The microsystem is also supposed to be compact and remotely actuated and therefore a certain structural complexity is required in order to have a reasonable number of design parameters. Therefore, excluding single-loop structures, two-loop topologies were selected. This choice of a KC with two independent loops, $L_{IND}=2$, was considered a good compromise between the need of maintaining the structural complexity under reasonable levels and the opportunity to have a great number of parameters available for design. Furthermore, a two-loop topology guarantees three anchored zones for the suspended parts.

As known, there are only two KC with six links only and one DoF, namely, Stephenson’s and Watt’s KCs, and the former was selected because its mobile ternary link floats, which usually gives a designer more opportunity to achieve a prescribed motion.

**Figure 5:**
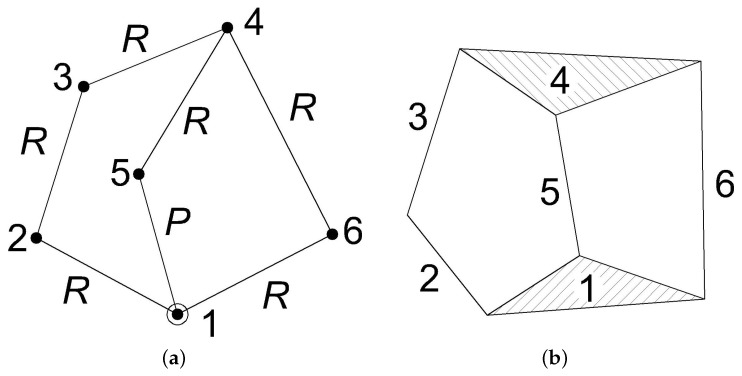
A representation of the Stephenson’s graph (**a**) and kinematic chain (**b**).

Figure 5a shows the graph representation of the Stephenson KC. Another possible way to define the topology of a KC is the polynomial representation illustrated in Figure 5b, according to which binary or ternary links are represented as lines or triangles, respectively.

With reference to Figure 5, one of the two ternary links (1 or 4) can be taken as the frame link (e.g., 1), while the floating binary link 3 can be conveniently used as the output link, that is, the link which will carry the operating lancet. The input link 5 was selected among the three links (2, 5 or 6) that are adjacent to the frame link 1.

Considered the required task, only revolute joints labels R were assigned to all the edges of the graph (kinematic pairs) except for input link 5, which was supposed to slide on the frame link.

The specific problem of synthesis consists in finding a plane mechanism which is able to drive the lancet tip according to a prescribed path, namely, parallel to the lumen wall, with the possibility of adjusting the ratio of the input by the output displacements.

### 2.8. Kinematic Synthesis

**Figure 6:**
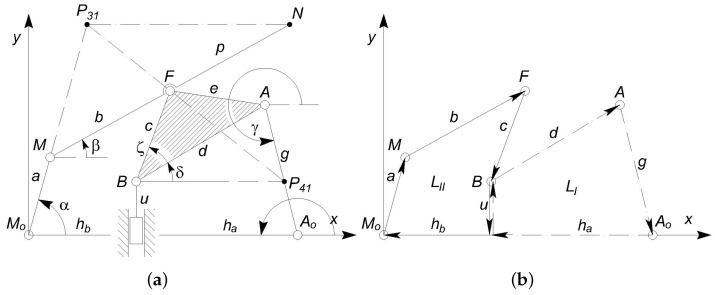
The nomenclature adopted to define the PRBM (**a**) and two independent vector loops $L_{I}$ and $L_{II}$ (**b**).

**Figure 7:**
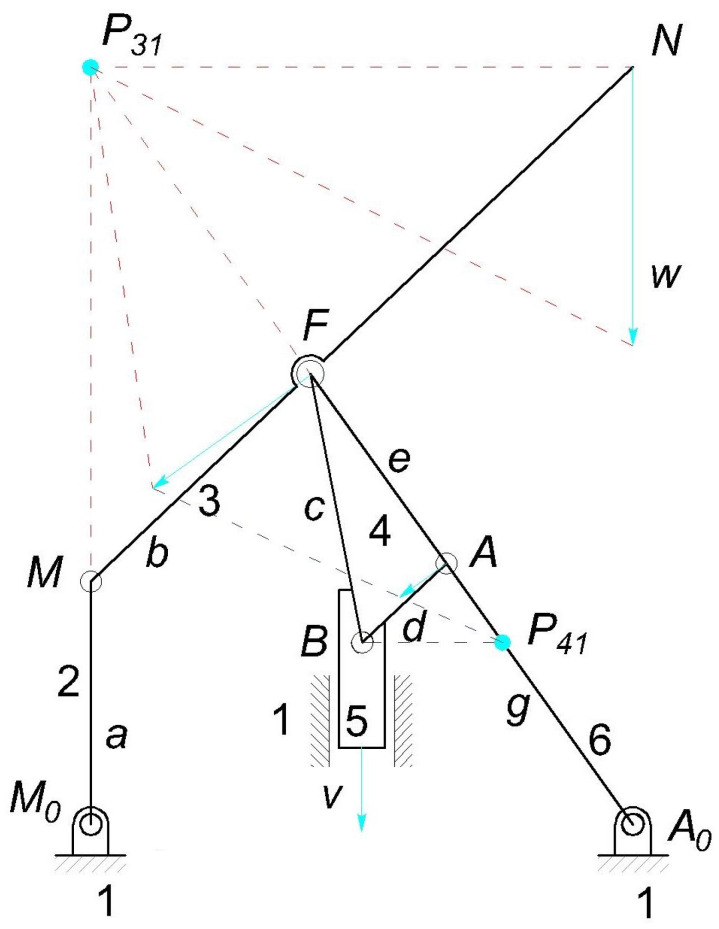
A view of the neutral configuration with the adopted nomenclature.

As a result of the topological stage, a mechanism with ordinary joint is obtained, as illustrated in Figure 6a, but the actual dimensions of the links have not been defined yet. In fact, these lengths are identified during the synthesis of the mechanism. The dimensions of the links and the neutral configuration can be calculated in order to make the tip point velocity ${\vec{v}}_{N}$ vertical, which guarantees that the mechanism tip *N*, in its working range, will never damage the lumen internal wall. This can be done by conveniently setting the positions of the centers of instantaneous velocity $P_{41}$ and $P_{31}$ of the links 4 and 3 with respect to the ground link 1. In fact, the line $P_{31}N$ can be oriented to be parallel to the *x*-axis, which also implies that velocity ${\vec{v}}_{N}$ will be parallel to the *y*-axis. Figure 7 illustrates geometrically how the positions of the centers $P_{31}$ and $P_{41}$ affect the output velocity $\vec{w}\equiv {\vec{v}}_{N}$ for a given input velocity $\vec{v}\equiv {\vec{v}}_{B}$.

Furthermore, the mechanical advantage ρ can be introduced as the mechanical amplification of the output force with respect to the input one. Assuming that no power is dissipated and the mechanism is working in static condition, and neglecting the force necessary to deflect the structure, this factor will be given by the ratio of the input velocity $\vec{v}$ of the actuator by the output velocity $\vec{w}$ of the tip. Considering the present application, the force could be either amplified (ρ>1) or reduced (ρ<1), provided that the elastic energy keeps negligible values. The mechanical advantage $\rho =\frac{v}{w}$ can be easily assessed geometrically from the design scheme illustrated in Figure 7 by combining the basic following kinematic relations:

(7) $$\begin{array}{cc} {\vec{v}}_{B} & \equiv \dot{u}{\vec{e}}_{y}\equiv \vec{v} \end{array}$$

(8) $$\begin{array}{cc} {\vec{v}}_{A} & =\vec{v}+{\vec{\omega }}_{4}\times \vec{BA}={\vec{\omega }}_{6}\times \vec{A_{0}A}={\vec{\omega }}_{4}\times \vec{P_{41}A} \end{array}$$

(9) $$\begin{array}{cc} {\vec{v}}_{F} & ={\vec{\omega }}_{4}\times \vec{P_{41}F}={\vec{\omega }}_{3}\times \vec{P_{31}F} \end{array}$$

(10) $$\begin{array}{cc} {\vec{v}}_{N} & \equiv \vec{w}={\vec{\omega }}_{3}\times \vec{P_{31}N} \end{array}$$

### 2.9. Kinematic Analysis

With reference to Figure 6a,b, the lengths of the links will be renamed as

(11) $$\begin{array}{cc} M_{0}M & =a\;,\;MF=b\;,\;AA_{0}=g\;,\;FN=p\;, \end{array}$$

(12) $$\begin{array}{cc} \;FB & =c\;,\;BA=d\;,\;FA=e \end{array}$$

while the other lengths $h_{a}$, $h_{b}$, *u*, the variable angles α, β, γ, δ, and the constant angle ζ have been introduced. By representing each link in the Argand–Gauss plane, the constant module vectors $\vec{u}\equiv iu$, $\vec{d}\equiv de^{i\delta }$, $\vec{g}\equiv ge^{i\gamma }$, ${\vec{h}}_{a}\equiv -h_{a}$, define the first vector loop $L_{I}$, whereas the second loop $L_{II}$ is identified by the vector chain $\vec{a}\equiv ae^{i\alpha }$, $\vec{b}\equiv be^{i\beta }$, $\vec{c}\equiv ce^{i\left(\delta +\zeta +\pi \right)}$, $\vec{u}\equiv -iu$, ${\vec{h}}_{b}\equiv -h_{b}$.

Since the kinematic chain of the structure under analysis consists of a classical Stephenson chain, Euler’s equation predicts

(13) $$L_{IND}=m-\ell +1=7-6+1=2\;,$$

where the numbers *m* and *ℓ* of kinematic pairs and links are 7 and 6, respectively. For plane [57] mechanisms, provided that they present no partial mobility [58], two independent vector equations can be written by means of which four unknown angles can be calculated, once the input displacement Δu is assigned.

#### 2.9.1. Position Analysis

Although the position analysis is a nonlinear problem, an analytic closed form solution was carried out for all the unknown variables. In fact, angles γ and δ are decoupled with respect to the other two unknowns α and β and therefore they can be obtained by using the first vector closure equation

(14) $$ui+de^{i\delta }+ge^{i\gamma }-h_{a}=0\;,$$

where the angle δ can be eliminated by summing up the squares of the real and imaginary components, which allows $d^{2}$ to be expressed as

(15) $$d^{2}=g^{2}-2\;\cos\left(\gamma \right)gh_{a}+2\;\sin\left(\gamma \right)gu+{h_{a}}^{2}+u^{2}\;.$$

This equation can be solved in γ by using the classical substitution

(16) $$\begin{array}{cc} \cos\left(\gamma \right) & =\frac{-t^{2}+1}{t^{2}+1} \end{array}$$

(17) $$\begin{array}{cc} \sin\left(\gamma \right) & =2\;\frac{t}{t^{2}+1} \end{array}$$

where $t=\tan\frac{\gamma }{2}$, which leads to the closed form solution

(18) $$\tan\frac{\gamma }{2}=\frac{2gu\pm \sqrt{-d^{4}+2d^{2}g^{2}+2d^{2}{h_{a}}^{2}+2d^{2}u^{2}-g^{4}+2g^{2}{h_{a}}^{2}+2g^{2}u^{2}-{h_{a}}^{4}-2{h_{a}}^{2}u^{2}-u^{4}}}{d^{2}-g^{2}-2gh_{a}-{h_{a}}^{2}-u^{2}}$$

Then, Equation (14) can be solved directly with respect to δ.

Considering the other independent loop equation

(19) $$ae^{i\alpha }+be^{i\beta }+ce^{i\chi }-ui-h_{b}=0\;,$$

where

(20) χ=δ+ζ+π,

it is possible to obtain:

(21) $$\tan\frac{\beta }{2}=\frac{B_{1}\pm \sqrt{B_{21}+B_{22}+B_{23}+B_{24}}}{A}\;,$$

where

$$\begin{array}{cc} A & =2\;\cos\chi bc+2\;\cos\chi ch_{b}+2\;\sin\chi cu+a^{2}-b^{2}-2\;bh_{b}-c^{2}-{h_{b}}^{2}-u^{2}\;,\\ B_{1} & =2\;\sin\chi bc-2\;bu\;,\\ B_{21} & =4\;c^{2}\cos^{2}\chi u^{2}-a^{4}-b^{4}-c^{4}-{h_{b}}^{4}-u^{4}+2\;a^{2}b^{2}+2\;a^{2}c^{2}+2\;a^{2}{h_{b}}^{2}+2\;a^{2}u^{2}\;,\\ B_{22} & =2\;b^{2}c^{2}+2\;b^{2}{h_{b}}^{2}+2\;b^{2}u^{2}-2\;c^{2}{h_{b}}^{2}-6\;c^{2}u^{2}-2\;{h_{b}}^{2}u^{2}-8\;\cos\chi \sin\chi c^{2}h_{b}\;u\;,\\ B_{23} & =-4\;\cos^{2}\chi c^{2}{h_{b}}^{2}+4\;\cos\chi c^{3}h_{b}+4\;\cos\chi c{h_{b}}^{3}+4\;\sin\chi c^{3}u+4\;\sin\chi cu^{3}\;,\\ B_{24} & =4\;\sin\chi c{h_{b}}^{2}u-4\;\cos\chi a^{2}ch_{b}-4\;\cos\chi b^{2}ch_{b}+4\;\cos\chi ch_{b}\;u^{2}-4\;\sin\chi a^{2}cu-4\;\sin\chi b^{2}cu\;. \end{array}$$

Finally, the angle α can be calculated directly from Equation (19). As known, the plus or minus signs in Equations (18) and (21) correspond to different assembly modes for the mechanism and therefore are theoretically correct. However, since our system consists of a compliant mechanism, it presents a neutral configuration with minimum potential energy with no externally applied forces. Hence, the actual solution will be the one which corresponds to the assembly that is compatible with an initial position that is coincident with the unloaded configuration.

##### Numerical Example

**Figure 8:**
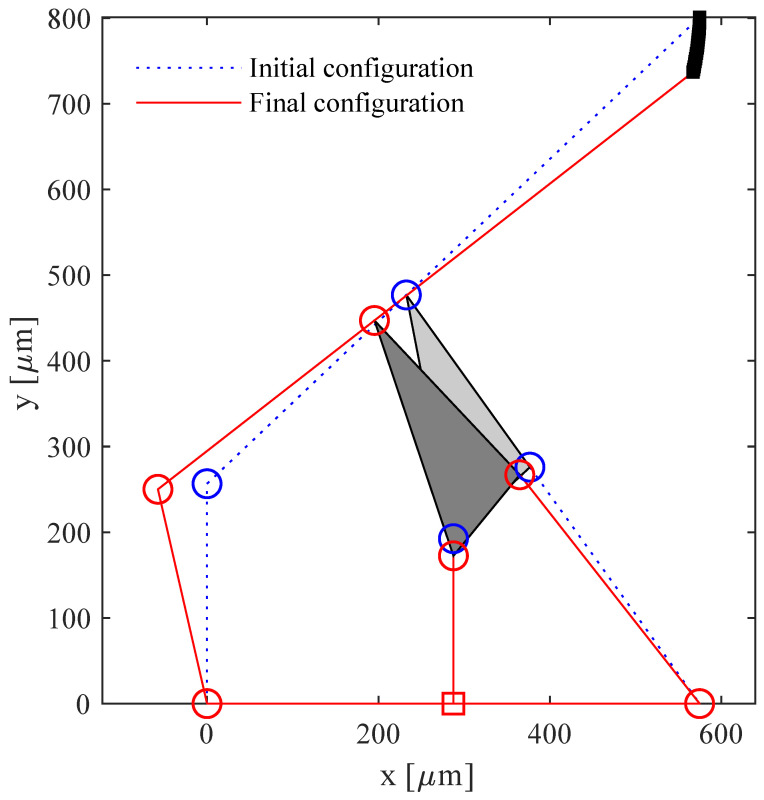
Initial and final configurations, corresponding to Δu=0 and Δu=−20 μm, respectively.

By using the parameters illustrated in Figure 6 and listed in Table 1, the motion of the PRBM was replicated for decreasing values of the input displacements Δu. Figure 8 shows the neutral and the deformed configurations obtained for displacements Δu from −1 to −20μm of point *B*.

#### 2.9.2. First Order Analysis

By multiplying by $e^{-i\gamma }$ the first derivative

(22) $$i\left(d\dot{\delta }e^{i\delta }+g\dot{\gamma }e^{i\gamma }+\dot{u}\right)=0$$

of the first vector loop equation (Equation (14)), and by extracting from the resulting equation

(23) $$i\left(d\dot{\delta }e^{i\left(\delta -\gamma \right)}+g\dot{\gamma }+\dot{u}e^{-i\gamma }\right)=0$$

its real part, $\dot{\gamma }$ is eliminated and so $\dot{\delta }$ can be obtained from

(24) $$-d\dot{\delta }\sin\left(\delta -\gamma \right)-\dot{u}\sin\gamma =0$$

as

(25) $$\dot{\delta }=\frac{\dot{u}\sin\gamma }{\sin\left(\delta -\gamma \right)d}\;.$$

Analogously, the other angular velocities

(26) $$\begin{array}{cc} \dot{\gamma } & =-\frac{\dot{u}\sin\delta }{\sin\left(\delta -\gamma \right)g} \end{array}$$

(27) $$\begin{array}{cc} \dot{\beta } & =\frac{\dot{u}\sin\alpha +c\dot{\delta }\sin\left(\delta -\alpha +\zeta \right)}{b\sin\left(\alpha -\beta \right)} \end{array}$$

(28) $$\begin{array}{cc} \dot{\alpha } & =-\frac{\dot{u}\sin\beta +c\dot{\delta }\sin\left(\delta -\beta +\zeta \right)}{a\sin\left(\alpha -\beta \right)} \end{array}$$

are found.

#### 2.9.3. Maximum Mechanical Advantage

It is worth noting that the mechanical advantage is a well known ratio in Mechanism Science, but only in 1998 was this concept extensively applied to compliant mechanisms [50,51]. The amplification force ratio ρ of the output to the input force can be easily calculated only at the cost of neglecting the overall energy $U_{O}$ required to take the compliant mechanism from its neutral position to its final configuration. Generally speaking, introducing the input and output forces ($f_{{}_{IN}}$ and $f_{{}_{OUT}}$) and displacements ($\delta_{{}_{IN}}$ and $\delta_{{}_{OUT}}$), from the energy balance

(29) $$f_{{}_{IN}}\delta_{{}_{IN}}=f_{{}_{OUT}}\delta_{{}_{OUT}}+U_{O}$$

the mechanical advantage can be expressed as

(30) $$\rho =\rho_{k}-\frac{U_{O}}{f_{{}_{IN}}\delta_{{}_{OUT}}}$$

where

(31) $$\rho =\frac{f_{{}_{OUT}}}{f_{{}_{IN}}}\;,$$

and

(32) $$\rho_{k}=\frac{\delta_{{}_{IN}}}{\delta_{{}_{OUT}}}$$

the latter representing the maximum value of the mechanical advantage obtained by neglecting $U_{O}$. This value can be obtained by using only kinematic equations, as described in the following lines.

The position, velocity and acceleration of the tip point *N* can be expressed as

(33) $$\begin{array}{cc} \vec{M_{0}N} & =ae^{i\alpha }+\left(b+p\right)e^{i\beta } \end{array}$$

(34) $$\begin{array}{cc} {\vec{v}}_{N} & =ia\dot{\alpha }e^{i\alpha }+i\left(b+p\right)\dot{\beta }e^{i\beta } \end{array}$$

(35) $$\begin{array}{cc} {\vec{a}}_{N} & =ia\ddot{\alpha }e^{i\alpha }-a{\dot{\alpha }}^{2}e^{i\alpha }+i\left(b+p\right)\ddot{\beta }e^{i\beta }-\left(b+p\right){\dot{\beta }}^{2}e^{i\beta } \end{array}$$

and so the mechanical advantage will be

(36) $$\rho_{k}=\sqrt{\frac{{\dot{u}}^{2}}{2a\left(b+p\right)\dot{\alpha }\dot{\beta }\cos\left(\alpha -\beta \right)+a^{2}{\dot{\alpha }}^{2}+{\left(b+p\right)}^{2}{\dot{\beta }}^{2}}}\;,$$

where $\dot{u}$ is given and the angular velocities $\dot{\alpha }$ and $\dot{\beta }$ were calculated (27) and (28).

### 2.10. Rigid-Body Replacement

**Figure 9:**
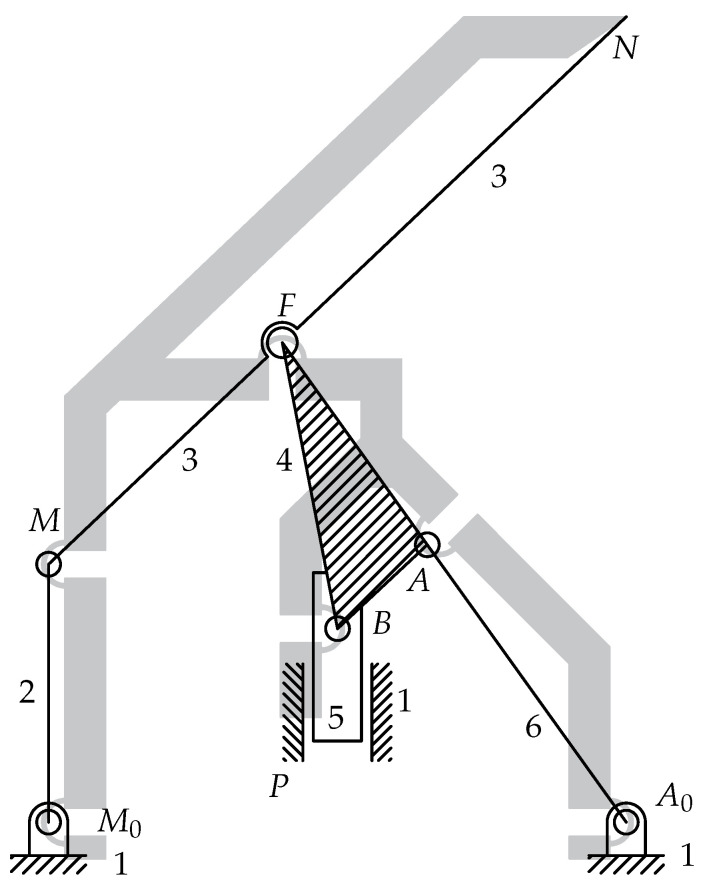
A view of the synthesized PRBM, overlapping with the compliant mechanism that is obtained after the joint replacement stage.

Figure 9 exemplifies the rigid-body replacement method. The centers $M_{0}$, *M*, *F*, *B*, *A*, and $A_{0}$ of the revolute joints R are replaced by elastic joints whose center of elastic weight is coincident with the elastic curved beams. The prismatic joint is not replaced, serving as input joint. The compliant mechanism is represented in gray, while the corresponding PRBM overlaps with the compliant mechanism. The figure shows four binary links, namely, 2 ($M_{0}M$), 3 (MF), 5 (the slider) and 6 ($A_{0}A$), and two ternary links, which are 1 (the frame link) and 4 (ABF).

### 2.11. Inverse Dynamic Analysis

**Figure 10:**
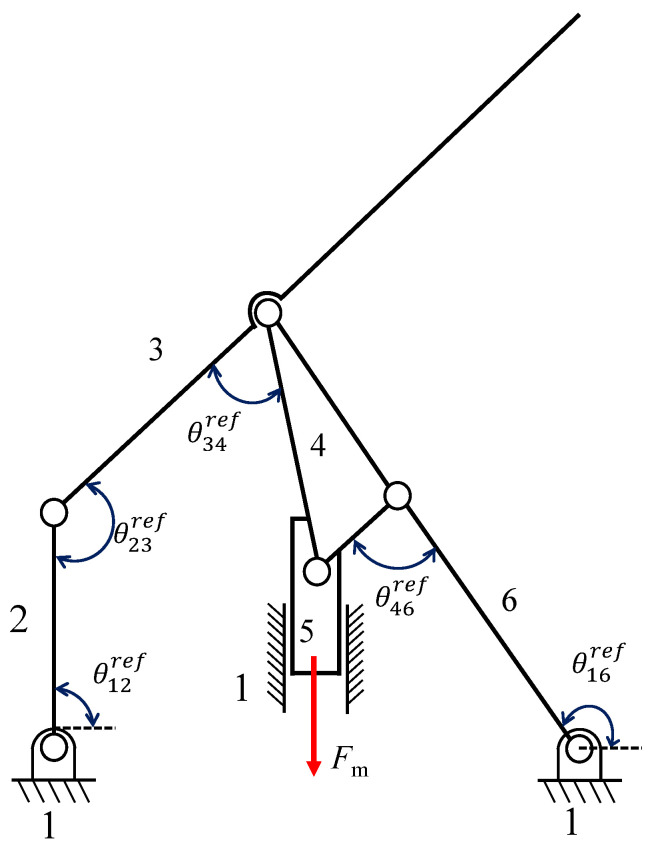
Reference angles with respect to the neutral position of the mechanism.

**Figure 11:**
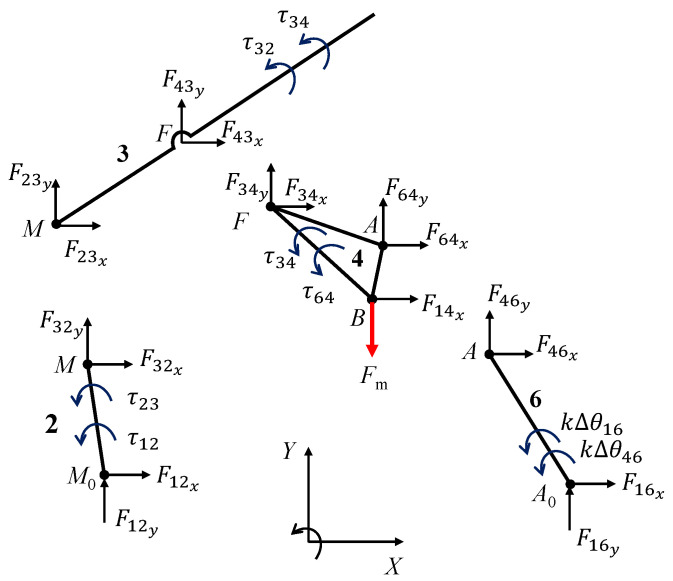
Illustration of the forces acting on the PRBM in a deformed position.

The stiffness of the curved beams was estimated assuming that the PRBM works in static condition and the friction in all kinematic pairs can be neglected. In the following analysis, the kinematic pair B is considered as a pin-in-a-slot, so it is assumed that the input force $F_{m}$ acts actually on link 4, as illustrated in Figure 10. The figure also shows the reference angles $\theta_{ij}^{ref}$. Under such circumstances, the free-body method can be exploited to write a system of 12 equations in 12 unknowns, keeping in mind that $F_{ij}=-F_{ji}$ and $\tau_{ij}=-\tau_{ji}$. The free-body diagram of the PRBM is reported in Figure 11. The system unknowns are the components of the reactions exerted by the kinematic pairs $F_{12_{x}},F_{12_{y}},F_{23_{x}},F_{23_{y}},F_{34_{x}},F_{34_{y}},$ $F_{46_{x}},F_{46_{y}},F_{14_{x}},F_{16_{x}},F_{16_{y}}$ and the stiffness constant *k*. Since the PRBM neutral and deformed positions are known, the angles $\Delta \theta_{ij}$ can be obtained (see also Section 2 and Figure 6a) as

$$\begin{array}{cc} \Delta \theta_{12} & =\theta_{12}-\theta_{12}^{ref}=\alpha -\alpha^{ref}\;,\\ \Delta \theta_{23} & =\theta_{23}-\theta_{23}^{ref}=\left(\beta -\beta^{ref}\right)-\left(\alpha -\alpha^{ref}\right)\;,\\ \Delta \theta_{34} & =\theta_{34}-\theta_{34}^{ref}=\left(\chi -\chi^{ref}\right)-\left(\beta -\beta^{ref}\right)\;,\\ \Delta \theta_{46} & =\theta_{46}-\theta_{46}^{ref}=\left(\gamma -\gamma^{ref}\right)-\left(\delta -\delta^{ref}\right)\;,\\ \Delta \theta_{16} & =\theta_{16}-\theta_{16}^{ref}=\left(\gamma -\gamma^{ref}\right)\;. \end{array}$$

Hence, denoting ψ=π−δ−ζ and introducing the system coefficients matrix $\left[A\right]$, the unknowns vector {X} and the constant terms vector {b},

$$\begin{array}{c} A=\left[\begin{array}{cccccccccccc} 1 & 0 & -1 & 0 & 0 & 0 & 0 & 0 & 0 & 0 & 0 & 0\\ 0 & 1 & 0 & -1 & 0 & 0 & 0 & 0 & 0 & 0 & 0 & 0\\ 0 & 0 & a\sin\alpha & -a\cos\alpha & 0 & 0 & 0 & 0 & 0 & 0 & 0 & \Delta \theta_{123}\\ 0 & 0 & 1 & 0 & -1 & 0 & 0 & 0 & 0 & 0 & 0 & 0\\ 0 & 0 & 0 & 1 & 0 & -1 & 0 & 0 & 0 & 0 & 0 & 0\\ 0 & 0 & 0 & 0 & b\sin\beta & -b\cos\beta & 0 & 0 & 0 & 0 & 0 & \Delta \theta_{234}\\ 0 & 0 & 0 & 0 & 1 & 0 & -1 & 0 & 0 & 0 & 1 & 0\\ 0 & 0 & 0 & 0 & 0 & 1 & 0 & -1 & 0 & 1 & 0 & 0\\ 0 & 0 & 0 & 0 & -c\sin\psi & c\cos\psi & d\sin\delta & -d\cos\delta & 0 & 0 & 0 & \Delta \theta_{346}\\ 0 & 0 & 0 & 0 & 0 & 1 & 0 & 1 & 0 & 0 & 0 & 0\\ 0 & 0 & 0 & 0 & 0 & 0 & 1 & 0 & 1 & 0 & 0 & 0\\ 0 & 0 & 0 & 0 & 0 & 0 & g_{1} & g_{2} & 0 & 0 & 0 & \Delta \theta_{461} \end{array}\right] \end{array}$$

$$\begin{array}{cc} {\left\{b\right\}}^{T} & =\left\{\begin{array}{cccccccccccc} 0 & 0 & 0 & 0 & 0 & 0 & 0 & F_{m} & 0 & 0 & 0 & 0 \end{array}\right\}\\ {\left\{X\right\}}^{T} & =\left\{\begin{array}{cccccccccccc} F_{12_{x}} & F_{12_{y}} & F_{23_{x}} & F_{23_{y}} & F_{34_{x}} & F_{34_{y}} & F_{46_{x}} & F_{46_{y}} & F_{16_{x}} & F_{16_{y}} & F_{14_{x}} & k \end{array}\right\} \end{array}$$

where $\Delta \theta_{123}=-\left(\Delta \theta_{12}-\Delta \theta_{23}\right)$, $\Delta \theta_{234}=-\left(\Delta \theta_{23}-\Delta \theta_{34}\right)$, $\Delta \theta_{346}=-\left(\Delta \theta_{34}-\Delta \theta_{46}\right)$, $\Delta \theta_{461}=-\left(\Delta \theta_{46}+\Delta \theta_{16}\right)$, $g_{1}=-g\sin\left(\gamma -\pi \right)$, $g_{2}=g\cos\left(\gamma -\pi \right)$, the static equilibrium equations can be written in matrix form as

(37) $$\left[A\right]\left\{X\right\}=\left\{b\right\}\;.$$

Therefore, the stiffness *k* can be obtained from the solution of the previous system.

**Figure 12:**
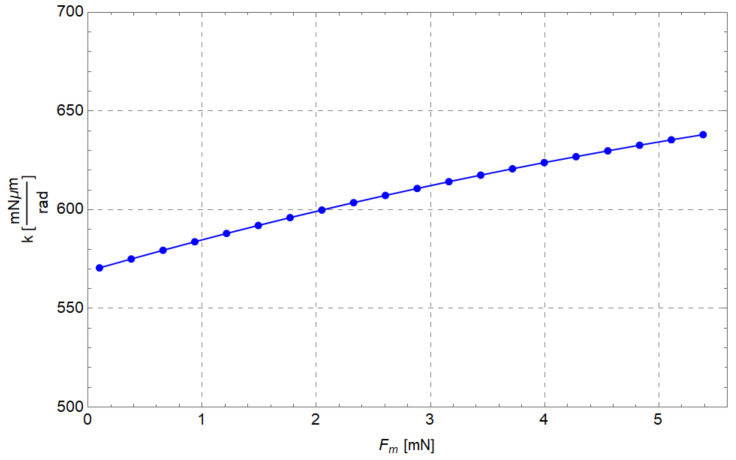
Curved beam stiffness (*k*) vs input force ($F_{m}$).

In Figure 12, the stiffness values obtained for 20 values of the input force ($F_{m}$) are reported. As can be seen, the stiffness slightly varies as the force increases. However, the minimum value of stiffness is 90% of the maximum value (638 mNμm/rad = $6.38\times 10^{-7}$ Nm/rad). Probably such behaviour is due to ill conditioning of the matrices, since the angular displacements ($\Delta \theta_{ij}$) are small. The stiffness ($5.65\times 10^{-7}$ Nm/rad) calculated with Equation (1) leads to an underestimation, confirming that an inverse dynamic analysis should be performed to accurately assess the stiffness of flexure elements.

### 2.12. Elastic Energy

The elastic energy stored in the PRBM can now be easily calculated from Equation (4) since the stiffness constant was estimated.

**Figure 13:**
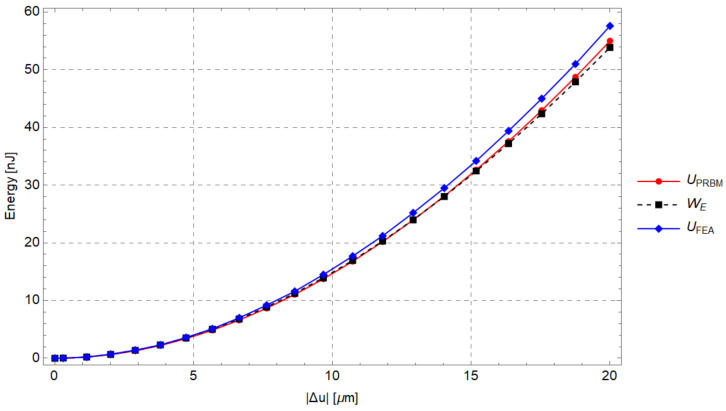
Comparison for increasing values of the input displacement among the strain energy of the compliant mechanism and the elastic energy of the PRBM.

For increasing values of the input force and its corresponding displacement, Figure 13 shows the elastic energy stored in the PRBM, the work of the external force ($W_{E}=\frac{1}{2}F_{m}\Delta u$) and the strain energy obtained by means of FEA.

In the following paragraph, FEA simulations will be performed to calculate the equivalent $k_{FEA}$ introduced in Section 2 and to check the PRBM validity.

### 2.13. Numerical Simulation

**Figure 14:**
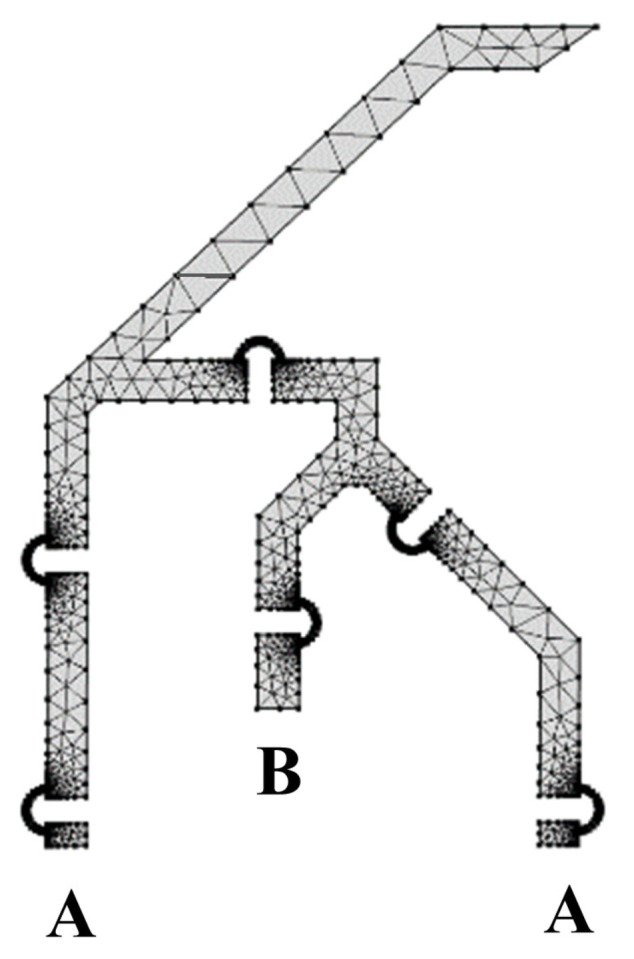
Finite element analysis set-up: fixed supports (**A**) and input force/displacement (**B**).

A set of finite element analyses were conducted with the commercial software Comsol Multiphysics^®^. The simulation set-up is represented in Figure 14: two fixed supports were introduced to model the anchored parts of the lancet (regions A), whereas a set of displacements were imposed to model the input action (region B). The generated mesh was composed of 1732 elements and refined for the flexible elements. Furthermore, cubic geometry shape function was selected and nonlinearity due to large deflections was considered. FEA simulations were carried out by implementing the 2D model of the device (30 μm out-of-plane thickness) to reduce the computational efforts. The device is made of Si [100] and the anisotropic formulation of elasticity was implemented in the FEM software. The elastic constant was inferred from [59] and is reported in Table 2 with other meaningful properties.

Firstly, twenty simulations were conducted by imposing displacements with magnitude from 1 μm to 20 μm, with steps of 1 μm, and direction opposite to the *y*-axis. The tip displacement (point *N*) was calculated for each simulation in order to compare it with the path obtained from the PRBM. The aim of this simulation was to asses the feasibility of the rigid-body replacement method. In fact, a good comparison would show that the compliant mechanism is able to replicate the behaviour of the PRBM and, therefore, to comply with the design requirements.

**Figure 15:**
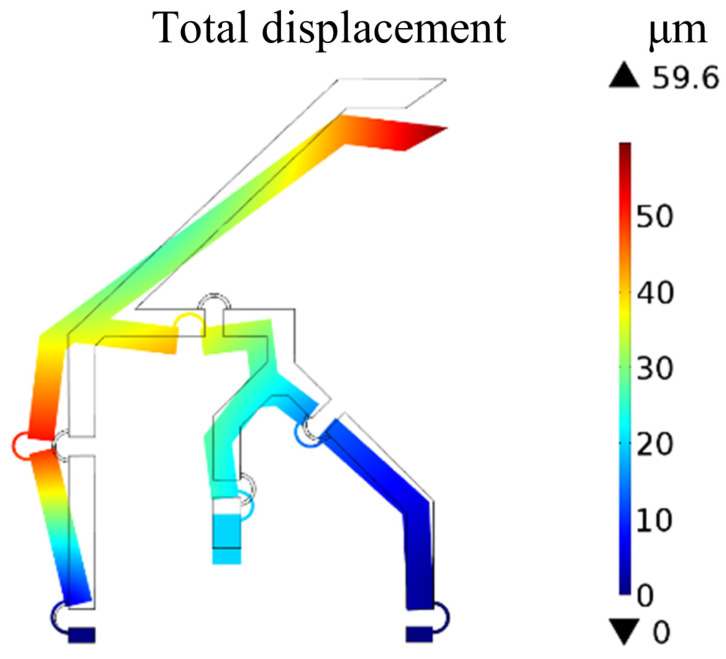
Finite Element Analysis of the compliant structure: neutral configuration (wireframe) and deformed one for Δu=−20 μm.

Figure 15 shows the neutral configuration of the compliant mechanism and the deformed one corresponding to the displacement with magnitude of 20 μm.

**Figure 16:**
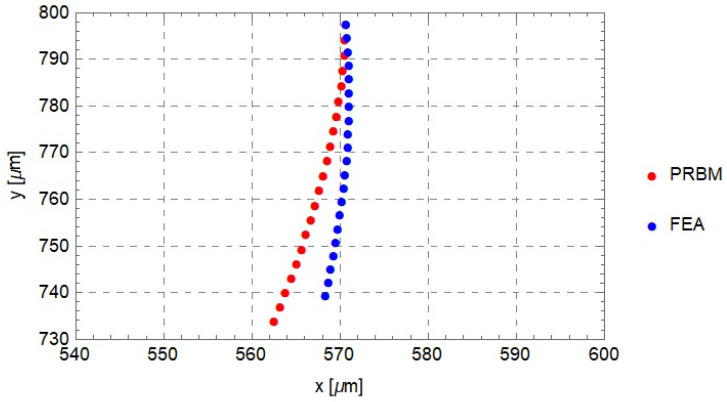
Tip displacements for the PRBM and for the corresponding compliant mechanism. Input displacements vary from Δu=−1μm to Δu=−20 μm.

Figure 16 represents the paths followed by the tip of the PRBM (analytical) and by the tip of the compliant mechanism (FEA). The results show good agreement, especially for small values of the applied displacement. In fact, for values of the applied displacement that tend to zero, the tip velocity has a predominant component along the *y*-axis.

Both the simulated and analytical paths bend toward the internal zone of the lumen and so they appear quite conservative with respect to the risk of damaging the lumen wall.

**Figure 17:**
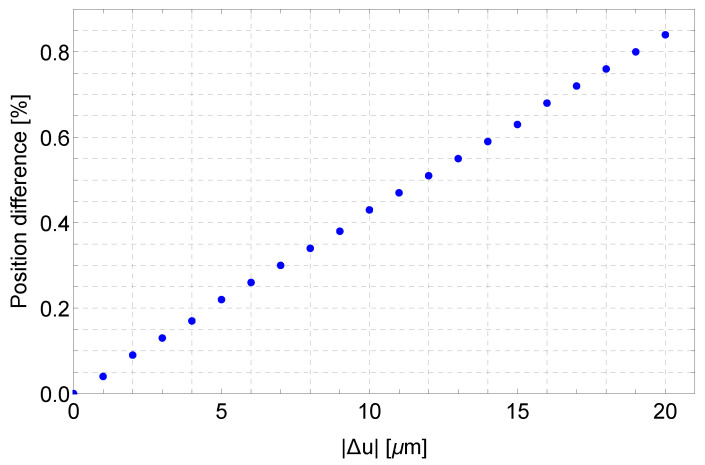
Percentage difference functions between the simulated and the analytical paths. Input displacements vary from Δu=−1μm to Δu=−20 μm.

The absolute displacement of the tip from the initial position can be calculated as the difference

$$\Delta \vec{M_{0}N}\left(t\right)=\vec{M_{0}N}\left(t\right)-\vec{M_{0}N}\left(t_{0}\right)\;,$$

where $t_{0}$ corresponds to the initial configuration. This vector can be calculated by using either FEA, obtaining the function $\Delta \vec{M_{0}N}{\left(t\right)}_{FEA}$, or by the analytical method, obtaining the function $\Delta \vec{M_{0}N}{\left(t\right)}_{SYM}$. Figure 17 shows, for the different applied displacements, the deviation

$$E_{T}\left(t\right)=\left|\Delta \vec{M_{0}N}{\left(t\right)}_{FEA}-\Delta \vec{M_{0}N}{\left(t\right)}_{SYM}\right|$$

of the magnitude of the position vector between the tip point of the PRBM and the tip point of the compliant mechanism $E_{T}\left(t\right)$, normalized with respect to the actual value of $\left|\vec{M_{0}N}\left(t\right)\right|$ and expressed as a percentage value.

These results show that deviation grows with the applied input displacement Δu. For example, in correspondence with Δu=−20μm, the deviation $E_{T\%}\left(t\right)$ is about 0.8 %. When the absolute deviation is considered not tolerable, for example, in the case of rotary comb drive actuation, a proper solution could be the adoption of CSFH hinges, which allow the compliant system to be very strictly related to the PRBM because the centers of the relative motion between the links always present the maximum limit equal to the conjugate surface gap (usually 1.5 μm).

Two additional simulations were carried out to evaluate the force transmission. In the first one, a force *F* was applied to region B, with magnitude 1.2 mN and direction opposite to the y-axis. To model the actuation action, a constraint was also applied to the displacements of the region (free translation along the y-axis and no translations allowed along the other axes). In this condition, the displacements of the actuator region and of the tip were obtained, as reported in Table 3. For an assigned time interval Δt, the mechanical advantage which can be calculated as the ratio between the input link displacement (*u*) along the y-axis divided by the tip displacement ($u_{{}_{OUT}}$) was equal to $\rho_{k}=0.34$.

In the second simulation, with respect to the setup described in the previous case, a force $F_{{}_{OUT}}$ was applied to the tip, with magnitude $F_{{}_{OUT}}=\rho_{k}F=$0.41 mN and direction concordant with the y-axis. As reported in Table 3, the displacement of the actuator region is negligible, whereas the tip displacement is about 2.5 μm.

The results of the second simulation, which are illustrated in Table 3, refer to a case of “isometric” exercise for which the effort exerted by the actuator is statically balanced by the force at the tip. The interesting result consists in the fact that by using a pair of input and output forces, whose ratio is coincident with the mechanical advantage, the input cable practically does not move, while the tip displacement is reduced from about −11μm to about a residual one that is equal to +2.5 μm, due to the curved beam compliance.

### 2.14. Stress Analysis

**Figure 18:**
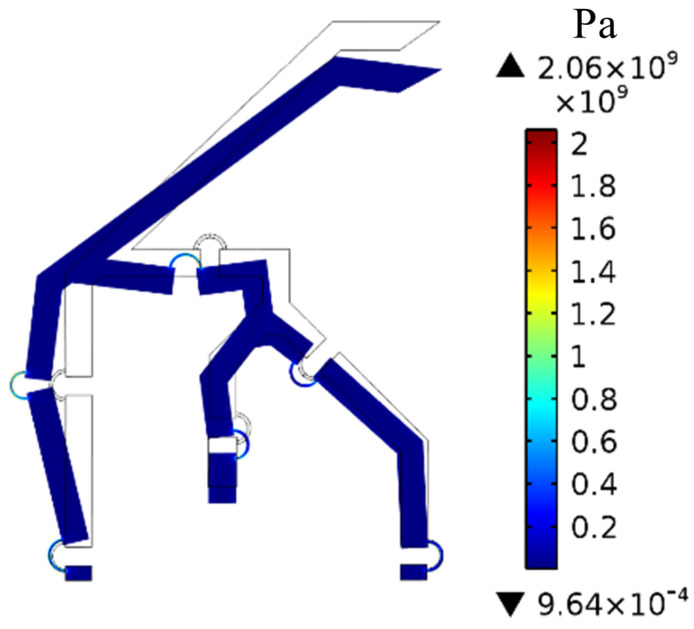
Distribution of the von Mises stress on the proposed compliant mechanism for the maximum input force ($F_{m}$ = 5.4 mN).

**Figure 19:**
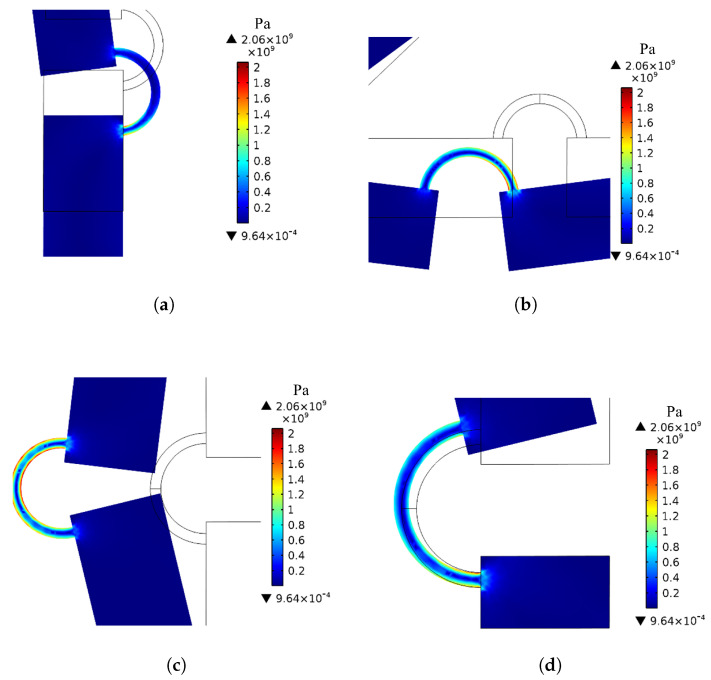
von Mises stress distribution on the most solicited flexure hinges for the maximum input force $F_{m}=$ 5.4 mN: (**a**) hinge *B*, (**b**) hinge *F*, (**c**) hinge *M*, (**d**) hinge $M_{0}$ (the labels in Figure 9 are used to designate flexure hinges).

The stress induced on the proposed compliant mechanism was verified for each loading condition considered in Section 3. Figure 18 illustrates the von Mises stress distribution on the entire mechanism for the maximum input force ($F_{m}$ = 5.4 mN). Figure 19a–d illustrate the stress distribution on the most solicited flexure hinges which are denoted as $M_{0},M,F,B$ in Figure 9. As might be expected, the stress is concentrated at the flexure hinges, where up to 2 GPa can be reached, while the other links undergo negligible stress. The ultimate tensile strength for Si-based materials should be verified experimentally once the fabrication process is complete since doping materials, etching technique, operating temperature, etc., affect the residual stresses. However, the ultimate tensile strength for Si (100), which is typically used in MEMS devices, may range from 1 to 4 GPa according to [60,61]. Since the residual stress resulting from a typical manufacturing process of a MEMS device may be as high as 0.5 GPa [62], the total mechanical stress is way below 4 GPa which falls within the tolerable range. As a matter of fact, the maximum tolerable stress should be experimentally evaluated and if the maximum stress calculated via FEA exceeds or is close to it, the cross section resistance of the flexure hinge should be increased, e.g., by increasing the out-of-plane thickness or its width. It should be kept in mind that if the width of the hinge is enlarged, the width of the rigid links should also be increased to ensure that elastic deformations are still concentrated in the flexure hinges.

## 3. Stiffness and Total Elastic Energy Estimation

**Figure 20:**
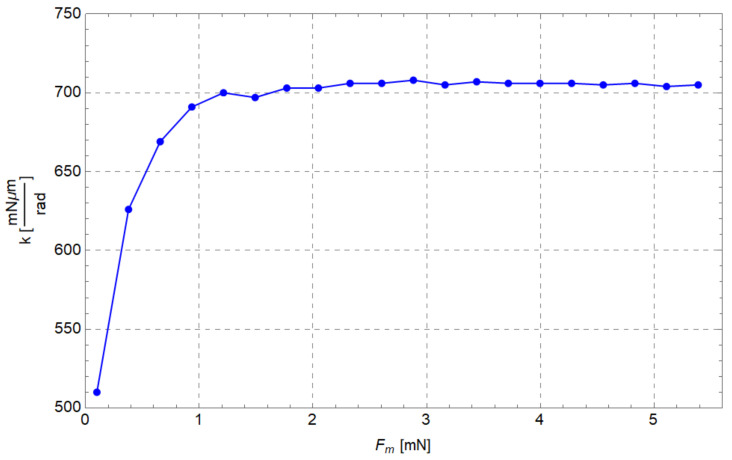
Curved beam stiffness vs input force obtained from FEA simulations.

Regarding the evaluation of the equivalent elastic constant of flexure hinges $k_{FEA}$, the following procedure was adopted. Region B was loaded by 20 input forces (from 0.1 mN to 5.4 mN) acting along the opposite direction of the y-axis; then the total elastic energy stored in the compliant mechanism $U_{FEA}$ was computed. Then, the angular displacement $\Delta \theta_{ij}$ was calculated for each curved beam by calculating the difference among the neutral and deformed configurations (see Figure 1). Then each $U_{FEA}$ was divided by the sum of the squares of $\Delta \theta_{ij}$ calculated for the corresponding configuration. Finally, an average $k_{FEA}=$$6.80\times 10^{-7}$ Nm/rad was so obtained. The plot of the stiffness obtained from FEA versus input force is shown in Figure 20. With respect to the inverse static analysis (see also Figure 12), it can be seen that $k_{FEA}$ is generally higher and remains approximately constant once $F_{m}$ = 0.9 mN is reached.

In Table 4, the average stiffnesses calculated by means of all the methods mentioned in this paper are summarized. The results are consistent with the remarks of Section 2.5. In fact, the stiffness evaluated by means of FEA is derived from the total energy $U_{O}$, which also takes into account the parasitic motion of the centers of the relative motion between two adjacent links. Furthermore, the stiffness calculated by means of Equation (1) leads to an underestimation of its actual value.

**Figure 21:**
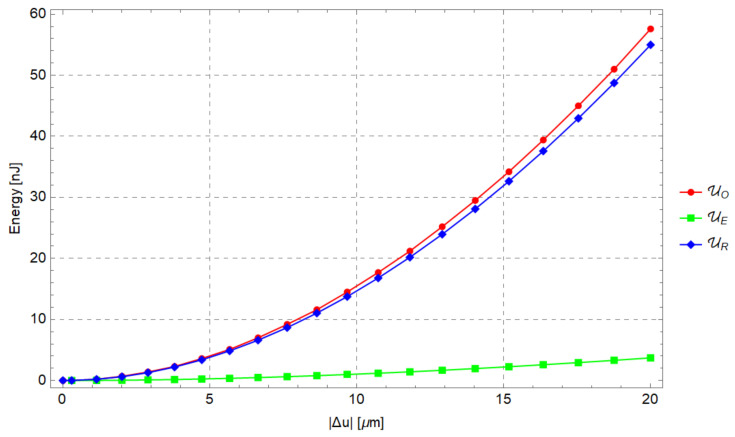
$U_{O},U_{R},U_{E}$ as a function of input displacement $\left|\Delta u\right|$, due to the input force $F_{m}$.

Figure 21 shows the comparison among the elastic energies evaluated by means of the analytical method ($U_{R}$), the numerical method ($U_{O}$) and the hybrid approach ($U_{E}$). The analytical predictions appear to be consistent with the numerical simulation results. It was found that the elastic energy $U_{E}$ is 6–8% of $U_{O}$, as can be seen in Figure 21. As a consequence, the energy $U_{E}$ may not be neglected *a priori* and it should be evaluated case by case when the PRBM is adopted to generate a compliant mechanism.

## 4. Further Developments

The next step for this project is the real construction of a prototype. This can be done on the basis of the previous experience by using DRIE on SOI wafer, as described in the previous sections. However there is still a feasible chance it can be implemented for improving positioning accuracy.

The main limitation for position accuracy of the method is intrinsically related to the joint replacement methods. In fact, the centers of the relative motions between any two adjacent links are not constant for the CM as they are for the PRBM. In fact, the latter presents classical revolute joints with fixed centers, while the CM presents flexure hinges composed of curved beams whose ends do not obey a rotary relative motion. Therefore, during the motion, the CM may present some configurations that are not fully consistent with the PRBM. By using curved beams as a flexure, the positioning error rarely exceeds 3%. In case a greater precision is required, there is the possibility of replacing the curved beams by the CSFH (Conjugate Surface Flexure Hinge) that have the portion of conjugate profiles that can control the errors over the position of the center of the revolute joint (see Refs. [38,55,56]).

## 5. Conclusions

A new alternative tool for atherectomy operations was presented in this paper. The development of this new device implied the construction of kinematic and dynamic modeling methods that could integrate the classical methods presented in the literature. Kinematic synthesis yielded a new pseudo-rigid-body equivalent mechanism (PRBM) that was transformed, by means of the joint replacement method, to a new compliant mechanism (CM). The latter was modeled by using a new approach to calculate the elastic energy stored in the elastic curved beams that were adopted as flexure hinges for the mechanism. Moreover, the new approach provided an analytical closed-form formulation of the mechanical advantage MA of the system. The method was validated by using FEA.

## 6. Patents

This paper presents a new concept microsystem that was registered at the Italian Patent Office on 26 June 2019. The article describes the characteristics of the new endoluminal system and shows possible ways to exploit this invention. More information concerning the above mentioned patent are available a the Italian Patent Office under the following registration number.

Belfiore, N.P., Ursi, P. Verotti, M., Remotely Actuated Lancet for Endoluminal treatments (in Italian), *Bisturi ad attuazione remota per trattamenti endoluminali*, Ufficio Italiano di Brevetti e Marchi (UBIM), Ministero dello sviluppo economico, Domanda Numero 102019000010158, 26 June 2019, property of University of Roma Tre, University of Roma “La Sapienza” and University of Genua.

Other related patents are the following.

- N.P. Belfiore, M. Scaccia, F. Ianniello, M. Presta, Selective Compliance Hinge, US 8,191,204 B2, 5 June 2012.
- N.P. Belfiore, M. Scaccia, F. Ianniello, M. Presta, Selective Compliance Hinge, World Intellectual Property Organization, WO 2009/034551 A1, Int. Appl. No. PCT/IB2008/053697, Publ. Date 19 March 2009.

## Figures and Tables

**Table 1:** Geometric parameters of the PRBM.

Parameter	Label	Value
$M_{0}M$	*a*	256.5 μm
MF	*b*	320.3 μm
FB	*c*	289.7 μm
BA	*d*	122.2 μm
FA	*e*	247.3 μm
$AA_{0}$	*g*	339.6 μm
	$h_{a}$	287.2 μm
	$h_{b}$	287.5 μm
	$u_{0}$	192.5 μm
$\hat{FBA}$	ζ	57.9 deg

**Table 2:** Material properties considered in the FEA simulations.

Property	Value	Unit
$E_{11}=E_{22}=E_{33}$	165.7	[GPa]
$E_{21}=E_{12}=E_{31}=E_{13}=E_{32}=E_{23}$	63.9	[GPa]
$E_{44}=E_{55}=E_{66}$	79.6	[GPa]
Density	2329	[kg/m${}^{3}$]
Poisson’s ratio	0.28	–

**Table 3:** Force transmission.

$F_{m}$ [mN]	*u* [μm]	$F_{{}_{\mathrm{OUT}}}$ [mN]	$u_{{}_{\mathrm{OUT}}}$ [μm]	ρ
1.20	−3.71	−−	−11.06	0.34
1.20	−0.06	−0.41	−2.46	−

**Table 4:** Average flexure element stiffness obtained by means of different methods. (ISA = Inverse Static Analysis).

Method	$k_{\mathrm{mean}}$ ($10^{-7}$ Nm/rad)
Equation (1)	5.65
ISA (Section 2)	6.07
FEA (Section 2.6)	6.80

## Data Availability

Not applicable.

## Nomenclature

The following nomenclatures are used in this manuscript:

*E*	Young’s modulus of the flexure hinge
*I*	second area moment of the flexure hinge cross-section about the bending axis
*L*	flexure hinge axis length
$k_{ij}$	stiffness of the generic flexure hinge that joins *i* and *j* links
$\tau_{ij}$	internal torques between link *i* and *j*
$\theta_{ij}^{ref}$	reference angle corresponding to the neutral position between link *i* and *j*
$\theta_{ij}$	angle corresponding to the deformed position between link *i* and *j*
$\Delta \theta_{ij}=\Delta \theta_{n}=\theta_{ij}-\theta_{ij}^{ref}$	angular displacement between deformed and neutral position for the *n*-th flexure hinge
$U_{O}$	overall energy for reaching a final deformed configuration
$U_{R}$	elastic energy due to the relative rotations between the rigid links
$U_{E}$	complementary energy quota that is adsorbed by the real compliant mechanisms due to the parasitic motion of the centers of the relative motion between two adjacent links
$\bar{\delta }=\vec{AB}-\vec{A^{\prime }B^{\prime }}$	displacement vector of the parasitic motion of the centers of the relative motion between two adjacent links (see Figure 3)
$F_{ij}=-F_{ji}$	reaction forces at the *i*-*j* revolute pair
$U_{FEA}$	elastic energy evaluated by means of FEA simulations
$k_{FEA}$	stiffness of a flexure hinge obtained via FEA simulations
$M_{0},M,F,B,A,A_{0}$	centers of the elastic joints
$L_{IND}$	independent loops of a kinematic chain
$\rho ,\rho_{k}$	mechanical advantage and maximum mechanical advantage
$\Delta u,u_{OUT}$	input and output displacement
$F_{m},F_{OUT}$	input and output force
$P_{41},P_{31}$	centers of instantaneous velocity of the links 4 and 3 with respect to the frame link 1
α,β,δ,γ,ζ,χ	angles for the kinematic analysis of the PRBM mechanism
